# Expression of the Orphan Cytosolic Sulfotransferase SULT4A1 and Its Major Splice Variant in Human Tissues and Cells: Dimerization, Degradation and Polyubiquitination

**DOI:** 10.1371/journal.pone.0101520

**Published:** 2014-07-02

**Authors:** Neelima P. Sidharthan, Neville J. Butcher, Deanne J. Mitchell, Rodney F. Minchin

**Affiliations:** Laboratory for Molecular and Cellular Pharmacology, School of Biomedical Sciences, University of Queensland, Brisbane, Australia; National University of Singapore, Singapore

## Abstract

The cytosolic sulfotransferase SULT4A1 is highly conserved between mammalian species but its function remains unknown. Polymorphisms in the SULT4A1 gene have been linked to susceptibility to schizophrenia. There are 2 major SULT4A1 transcripts in humans, one that encodes full length protein (wild-type) and one that encodes a truncated protein (variant). Here, we investigated the expression of SULT4A1 in human tissues by RT-PCR and found the wild-type mRNA to be expressed mainly in the brain, gastrointestinal tract and prostate while the splice variant was more widely expressed. In human cell-lines, the wild-type transcript was found in neuronal cells, but the variant transcript was expressed in nearly all other lines examined. Western blot analysis only identified SULT4A1 protein in cells that expressed the wild-type mRNA. No variant protein was detected in cells that expressed the variant mRNA. Ectopically expressed full length SULT4A1 protein was stable while the truncated protein was not, having a half-life of approximately 3 hr. SULT4A1 was also shown to homodimerize, consistent with other SULTs that contain the consensus dimerization motif. Mutation of the dimerization motif resulted in a monomeric form of SULT4A1 that was rapidly degraded by polyubiquitination on the lysine located within the dimerization motif. These results show that SULT4A1 is widely expressed in human tissues, but mostly as a splice variant that produces a rapidly degraded protein. Dimerization protects the protein from degradation. Since many other cytosolic sulfotransferases possess the conserved lysine within the dimerization motif, homodimerization may serve, in part, to stabilize these enzymes *in vivo*.

## Introduction

The cytosolic sulfotransferases are a superfamily of enzymes that catalyze the transfer of a sulfonate group from the common cofactor 3′-phosphoadenosine 5′-phosphosulfate to a wide range of endogenous and exogenous substrates [1], [2]. In humans, there are 3 known families based on sequence identity (SULT1, SULT2 and SULT4). The only member of the SULT4 family is SULT4A1, which shares less than 40% sequence homology with other human sulfotransferases but is highly conserved between mammalian species [3]. SULT4A1 was originally isolated from brain tissue [4] and studies in mice indicate that it is primarily expressed in that tissue [5]. Moreover, it exhibits sex-dependent expression, with mRNA levels approximately 4-fold higher in female animals [6].

**Figure 1:**
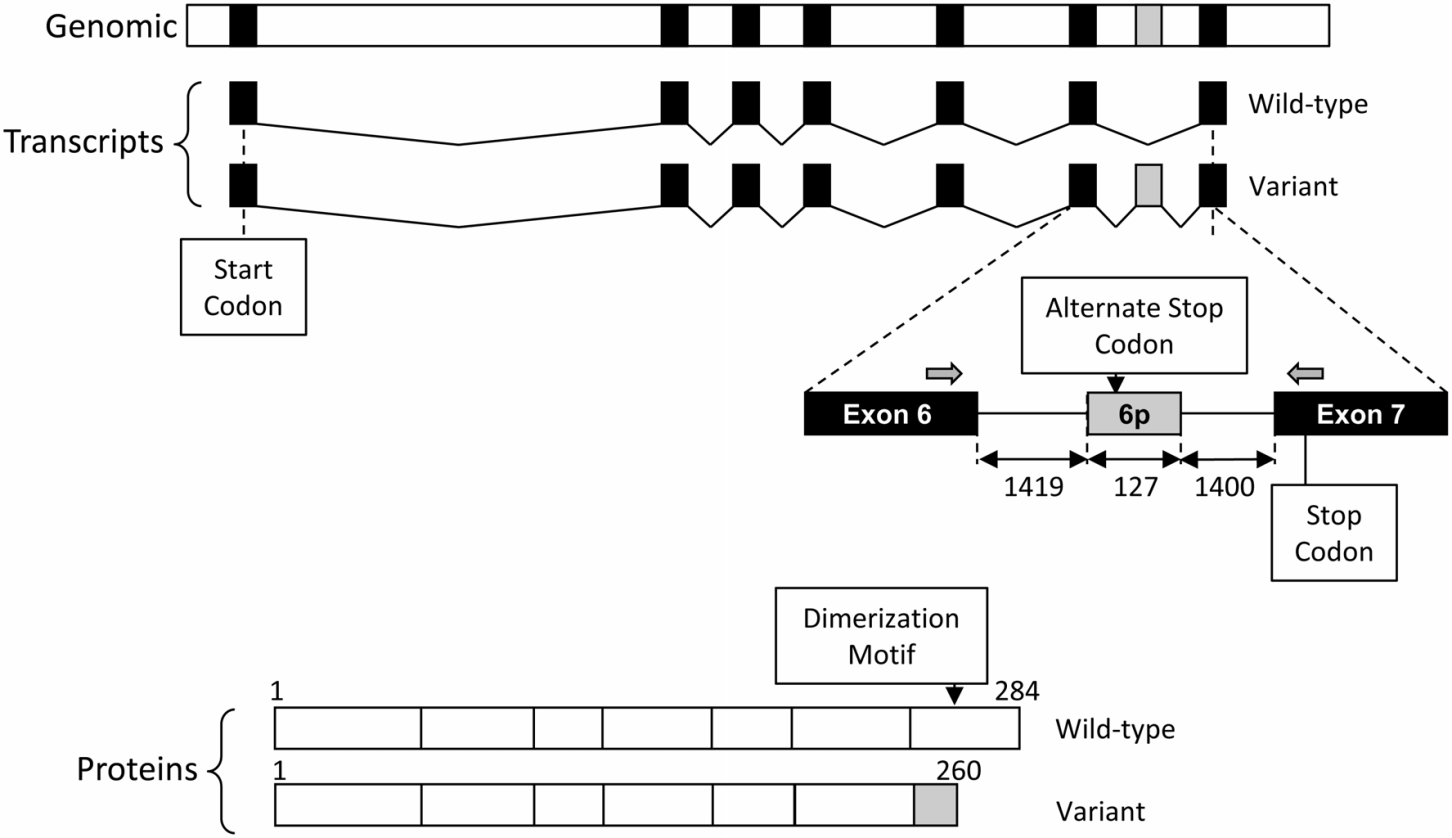
Line-box diagram showing the exon-intron arrangement for wild-type and variant SULT4A1 transcripts. Black boxes represent exons while the grey box represents part of the intron between exon 6 and 7 that is incorrectly spliced in the variant. The positions of the PCR primers are denoted by the grey arrows. The resulting SULT4A1 proteins are also shown. The diemerization motif, which is located in exon 7, is absent in the variant protein.

SULT4A1 gene expression is regulated by CREB and ATF-2, which bind to conserved sites within 100 bp of the major transcription start site [7]. There are 2 major transcripts, one that encodes the complete protein sequence and a second that introduces a premature stop codon due to the failure to correctly excise the intron between exons 6 and 7 (Fig. 1) [4]. The second transcript predicts a truncated protein, although it has been suggested the mRNA is unstable and subject to nonsense mediated mRNA decay [3]. The SULT4A1 protein is post-translationally modified by phosphorylation involving ERK1/2 [8]. This introduces a motif that is identified by the prolyl cis-trans isomerase Pin1 to destabilize the protein. Despite our understanding of SULT4A1 regulation, no substrate for the enzyme has been identified to date and its exact role in the brain remains unknown. Interestingly, a number of studies have demonstrated an association between specific single nucleotide polymorphisms in the SULT4A1 gene and susceptibility to schizophrenia [9], [10], [11].

In the present study, we have investigated two aspects of the SULT4A1 gene. Firstly, expression of the major SULT4A1 transcripts was examined in human tissues and cells. Secondly, the stability of the SULT4A1 protein products was determined. The results suggest that the full length protein is mostly restricted to neuronal cells. Similar to other sulfotransferases, SULT4A1 undergoes dimerization, which protects the protein from polyubiquitination and degradation by masking lysine 254, the site of polyubiquitination. A truncated SULT4A1 protein produced from the variant transcript was not able to homodimerize and was highly unstable.

## Materials and Methods

### Cell culture

All cell-lines used in this study were obtained from the American Type Culture Collection (Manassas, VA). SH-SY5Y were maintained in Advanced DMEM/F12 supplemented with 10% fetal bovine serum (FBS), SK-N-MC were in Advanced MEM with 10% FBS, PC-12 were in RPMI 1640 with 5% FBS and 10% heat-inactivated horse serum, and Neuro 2A, PC-3 and SKOV3 were in DMEM with 10% FBS. All other cell-lines were in RPMI 1640 supplemented with 10% FBS. All cells were maintained at 37°C in a humidified atmosphere of 5% CO_2_ in air. Media and supplements were from Life Technologies (Carlsbad, CA). Retinoic acid (Sigma-Aldrich, St. Louis, MO; 10 µM) was used to differentiate SK-N-MC cells as described previously [12].

### Site-directed mutagenesis and cloning

The generation of FLAG-tagged and HA-tagged human wild-type SULT4A1 plasmids (pFLAG-SULT4A1-WT and pHM6-SULT4A1-WT, respectively) has been described previously [13]. To generate the FLAG-tagged human variant SULT4A1 plasmid (pFLAG-SULT4A1-VAR), the same primers and procedure were used as in Mitchell et al [13] except that the template for the PCR was cDNA from IMR-32 cells, which only express the variant SULT4A1 transcript. The pFLAG-SULT4A1-TV>AE and pFLAG-SULT4A1-KTV>QAE mutants were generated by the GENEART site-directed mutagenesis system (Life Technologies) using pFLAG-SULT4A1-WT as template and the following forward and reverse primers; FP-TV>AE, 5′-GGAAGGACA-TCTTCGCCGAGTCCATGAATGAGAAG-3′, RP-TV>AE 5′-CTTCTCATTCATGG-ACTCGGCGAAGATGTCCTTCC-3′, FP-KTV>QAE 5′-AGAGTTGGGCTGTGGGA-GGACATCTTC-3′, RP-KTV>QAE 5′-GAAGATGTCCTCCCACAGCCCAACTCT-3′, respectively. Clone sequences were verified by sequencing.

### Cell transfection

IMR-32 cells were seeded in 6-well plates at 1×10^6^ cells per well. Cells were allowed to adhere overnight and then transfected with a total of 4 µg of plasmid DNA using LipofectAMINE 2000 reagent (Life Technologies) according to the manufacturer’s instructions. For mRNA and protein stability studies, cells were transfected with 4 µg of pFLAG-SULT4A1-WT, pFLAG-SULT4A1-VAR, pFLAG-SULT4A1-TV>AE or pFLAG-SULT4A1-KTV>QAE for 24 hr. For co-immunoprecipitation experiments, cells were co-transfected with 2 µg pHM6-SULT4A1-WT and either 2 µg pFLAG empty vector, pFLAG-SULT4A1-WT or pFLAG-SULT4A1-TV>AE for 24 hr. For polyubiquitination experiments, cells were transfected with 1 µg pcDNA-HA ubiquitin and 3 µg pFLAG-SULT4A1-WT, pFLAG-SULT4A1-VAR, pFLAG-SULT4A1-TV>AE or pFLAG-SULT4A1-KTV>QAE for 24 hr.

### Western blot analysis

Cells were washed with phosphate buffered saline (PBS), lysed in RIPA buffer (1% NP-40, 50 mM Tris [pH 7.4], 150 mM NaCl, 0.5% sodium deoxycholate) containing protease inhibitor cocktail (Roche, Mannheim, Germany) for 30 min on ice and then centrifuged at 4°C for 15 min at maximum speed. Protein concentration was determined by the method of Bradford [14] Samples were boiled in SDS reducing buffer, and 30 µg electrophoresed on 12% polyacrylamide gels and transferred to nitrocellulose membranes. Membranes were blocked with 5% non-fat milk in PBS containing 0.05% Tween-20 and then immunoblotted with anti-SULT4A1 primary antibody (Protein Tech Group, Chicago, IL) or anti-α-tubulin primary antibody (Cell Signaling Technology, Boston, MA) followed by anti-rabbit or anti-mouse HRP conjugated secondary IgG (Jackson ImmnoResearch, West Baltimore, PA), respectively. FLAG-tagged proteins were immunoblotted with anti-FLAG M2 HRP conjugated antibody (Sigma-Aldrich). Proteins were visualized using Western Lightning Plus-ECL (PerkinElmer, VIC, Australia) and a Kodak image station 4000 MM (Carestream Health, Rochester, NY).

### Co-immunoprecipitation studies

Following transfection as described above, IMR-32 cells were washed twice with cold PBS and then incubated on ice for 15 min in 0.8 ml cell lysis buffer (1% Triton X-100, 50 mM Tris [pH 7.4], 150 mM NaCl, 1 mM EDTA) containing protease inhibitor cocktail (Roche). Lysates were centrifuged for 10 min at maximum speed (4°C) and then 0.3 ml was transferred to duplicate tubes. Anti-HA antibody (Sigma-Aldrich, 1/200) was added to one of the duplicate tubes and then they were rotated at 4°C for 2 hr. Protein A agarose beads (Cell Signaling Technology) were added to each tube and then rotated for a further 1 hr at 4°C. The beads were then centrifuged for 30 s at 6000×*g*, supernatants discarded, and the beads washed 3 times with 0.5 ml of cell lysis buffer minus Triton X-100. Beads were then boiled in 50 µl SDS reducing buffer and Western blotted as described above.

### Polyubiquitination studies

After overnight transfection as described above, IMR-32 cells were treated with MG132 (20 µM, Sigma-Aldrich) or vehicle (DMSO) up to 8 hr, then washed twice with cold PBS and then incubated on ice for 15 min in 0.8 ml cell lysis buffer containing protease inhibitor cocktail (Roche). Lysates were centrifuged for 10 min at maximum speed (4°C) and supernatants transferred to fresh tubes. Anti-FLAG-M2 antibody conjugated agarose beads (Sigma-Aldrich) were added and the supernatants rotated at 4°C for 2 hr. Immunoprecipitates were collected by centrifugation for 30 s at 6000×*g* and the beads washed 3 times with 0.5 ml cell lysis buffer minus Triton X-100. Beads were then boiled in 50 µl SDS reducing buffer and Western blotted as described above. This experiment was also performed under denaturing conditions where 1% SDS was substituted for Triton X-100 in the cell lysis buffer, and the samples heated at 95°C for 10 min prior to the addition of anti-FLAG-M2 antibody conjugated agarose beads.

### Extraction of total RNA and cDNA synthesis

Total RNA was extracted from cell-lines using the RNAeasy mini kit (Life Technologies) according to the manufacturer’s instructions. This included an on-column DNAse treatment.Then cDNA was synthesized using 5 µg of total RNA and SuperScript II (Life Technologies) as described in the manufacturer’s protocol. Reactions lacking reverse transcriptase confirmed a lack of genomic DNA contamination. RNA from various human tissues was obtained from the FirstChoice Human Total RNA Survey Panel (Life Technologies), and cDNA was synthesized using 1 ug of RNA as above. FirstChoice Human Total RNA Survey Panel is undergoes a stringent DNAse treatment and is certified to be free of genomic DNA.

### Expression of SULT4A1 transcripts

Primers were designed to amplify a region spanning exons 5 to 7 of the *SULT4A1* gene. The PCR product for the wild-type transcript is 270 bp in length while the variant gives a product of 397 bp because the intron between exons 6 and 7 is not spliced out. The forward primer sequence was 5′-CTACGGCTCCTGGTTTGAG-3′ and the reverse primer sequence was 5′-ATGGAGACGGTGAAGATGTC-3′. These primers detected human, mouse and rat transcripts. The forward primer sequence for human β-actin was 5′-CCTCGCCTTTGCCGATCC-3′ and the reverse primer was 5′-GGATCTTCATGAGGTAGTCAGTC-3′. The forward primer sequence for mouse β-actin was 5′-CCTAAGGCCAACCGTGAAAAG-3′ and the reverse primer was 5′-TCTTCATGG TGCTAGGAGCCA-3′. PCR products were amplified from 1 µl cDNA template using AmpliTaq Gold (Life Technologies) in a final volume of 10 µl. Cycling conditions for both SULT4A1 and β-actin were 95°C for 10 min, followed by 40 cycles of 95°C for 10 s, 58°C for 10 sec, and 72°C for 30 s (SULT4A1) or 45 s (β-actin). PCR products were run on 2% agarose gels at 100 V for 45 min.

### Protein stability studies

To determine protein stability, transfected IMR-32 cells were treated with 10 µg/ml cycloheximide (Sigma-Aldrich) and incubated for 0, 2, 4, 6 and 8 h. At each time point, cells were prepared for Western blot as described above. Densitometry was performed using Adobe Photoshop CS4 software and results were normalized to α-tubulin and then expressed relative to the zero time point.

### Data analysis

All experiments were performed in triplicate. Data are expressed as mean ± SEM. Statistical comparisons between different groups were assessed by Student’s *t*-tests or one-way ANOVA assuming significance at *P*<0.05 using Prism 5 (GraphPad Software, San Diego, CA).

## Results

### Tissue and Cell expression of SULT4A1

To detect the presence of SULT4A1 transcripts in human tissues, mRNA was isolated and amplified by PCR. Primers were designed from exon 6 to exon 7 so that the wild-type transcript and the splice variant were detected in the same reaction. The wild-type transcript produced a PCR product of 270 bp while the variant produced a PCR product of 397 bp (Fig. 1).

**Figure 2:**
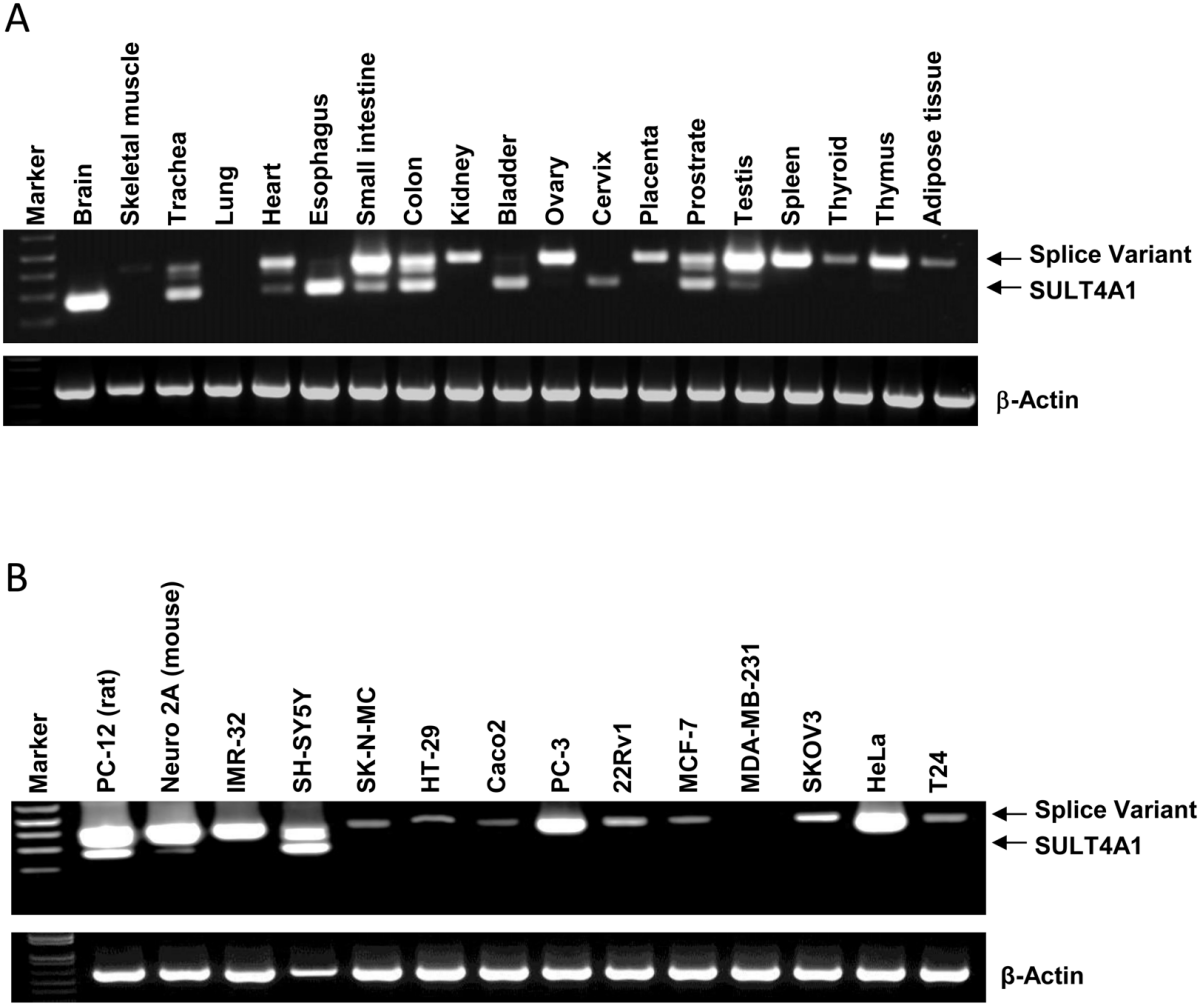
Expression of SULT4A1 transcripts in human tissues and cells. (A) mRNA from various human tissues was subjected to RT-PCR using primers that amplify both the wild-type (lower band) and variant (upper band) SULT4A1 transcripts. β-Actin was used as a control. (B) mRNA from various human and rodent cell-lines was amplified by RT-PCR.

Both the wild-type and the splice variant transcripts were observed in a variety of human tissues (Fig. 2A). The wild-type transcript was seen in the brain and gastrointestinal tract, similar to that reported for mice [5]. It was also observed in bladder, cervix and prostrate. The variant transcript was more common and was found in all tissues except the brain, lung and cervix. Several tissues such as the small intestine, colon and prostate showed the presence of both transcripts. Because of the heterogeneity of these tissues, it is not possible by RT-PCR to determine whether these transcripts are expressed in the same cell-types.

**Figure 3:**
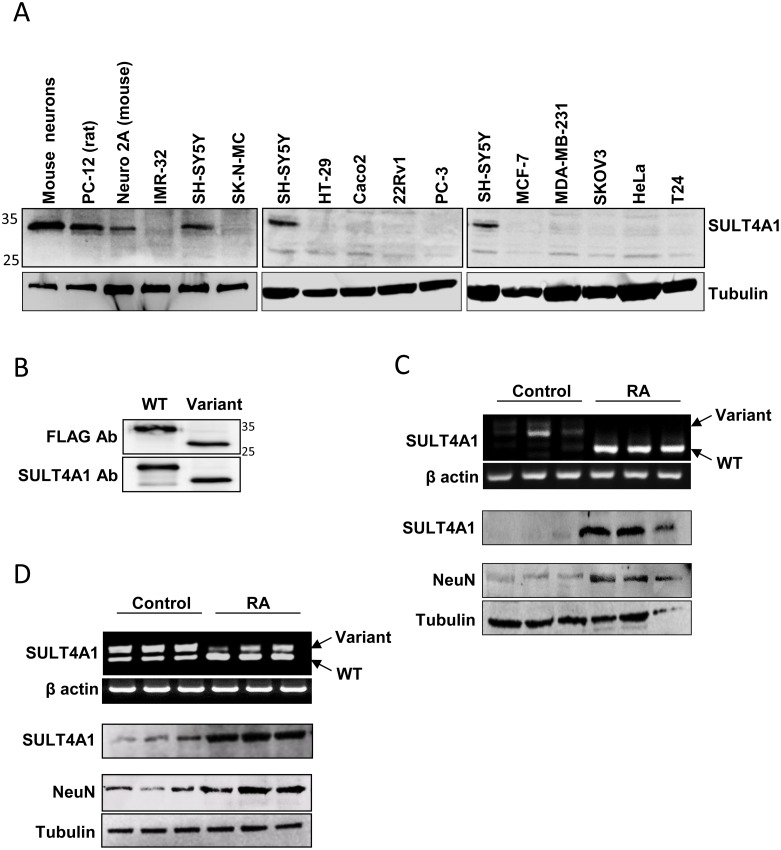
Expression of SULT4A1 protein. (A) SULT4A1 protein in various human and rodent cell-lines detected by Western blot. SH-SY5Y cells were used as a positive control on the three separate gels. Tubulin was included as a positive control. (B) Ectopically expressed FLAG-tagged wild-type (WT) and truncated (Variant) proteins were detected in IMR-32 cells by the polyclonal SULT4A1 antibody used in A. (C) Expression of SULT4A1 mRNA (upper panel) and SULT4A1 protein (middle panel) in SK-N-MC cells differentiated with retinoic acid (RA). (D) Expression of SULT4A1 mRNA (upper panel) and SULT4A1 protein (middle panel) in SH-SY5Y cells differentiated with retinoic acid (RA). NeuN protein is included as a neuronal marker. β-Actin was used as a control for the RT-PCR while tubulin was used as a control for the Western blots.

We next examined SULT4A1 expression in several human and rodent cell-lines derived from different tissues (Fig. 2B). While the splice variant was seen in most of the cell-lines, the wild-type transcript was only observed in rat PC-12, mouse Neuro 2A and human SH-SY5Y cells, all of which are derived from the neuronal tissue. When the different cell-lines were probed for the presence of SULT4A1 protein by Western blots, only those cells with the wild-type transcript expressed protein (Fig. 3A). The lack of any SULT4A1 in cells that expressed the splice variant was not due to the inability of the antibody to detect the resulting truncated protein from that transcript. Recombinant SULT4A1 expressed from either the wild-type or splice variant mRNA was detected by the antibody (Fig. 3B).

Human brain tissue specifically expressed wild-type SULT4A1 transcript while the undifferentiated neuronal cell lines SK-N-MC and IMR-32 only expressed the splice variant. To determine whether this difference might be related to the level of differentiation, SK-N-MC cells were treated with retinoic acid for 10 days. Differentiation was confirmed by the up-regulation of the neuronal marker NeuN (Fig. 3C). Upon differentiation, the SK-N-MC cells decreased the level of splice variant but increased the level of wild-type transcript that was expressed (Fig. 3C). In addition, there was increased expression of the SULT4A1 protein. This switch between transcripts and subsequent up-regulation of SULT4A1 protein was not dependent on retinoic acid as similar results were obtained following treatment of SK-N-MC cells with the differentiating agent bromodeoxyuridine (data not shown). Moreover, similar results were seen in SH-SY5Y cells differentiated with retinoic acid (Fig. 3D), where there was a switch from the variant to wild-type mRNA and a subsequent increase in SULT4A1 protein.

### Stability of SULT4A1 Proteins

**Figure 4:**
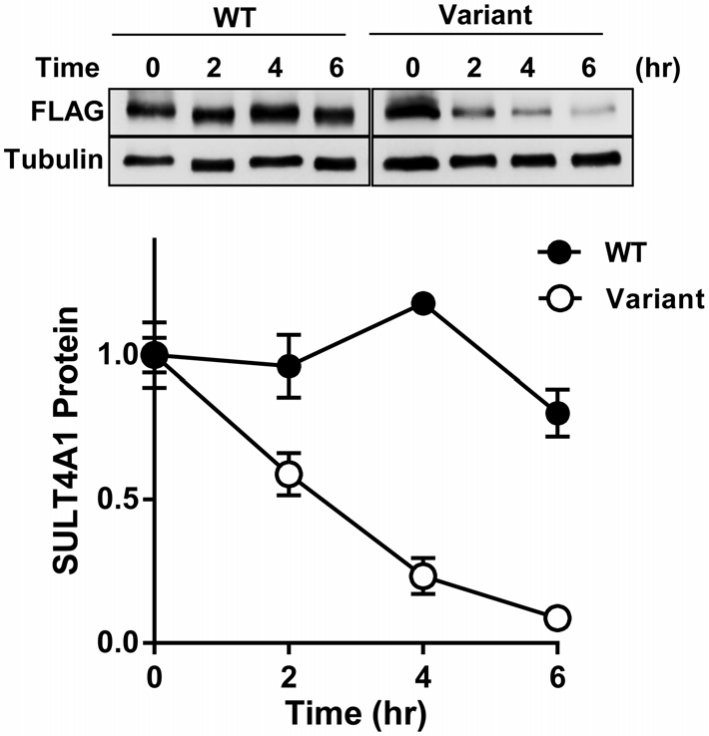
Stability of SULT4A1 protein in IMR-32 cells. Cells were transfected with plasmids for FLAG-tagged wild-type or variant SULT4A1 and then treated with 10 µg/ml cycloheximide for the indicated times. Proteins were then Western blotted using anti-FLAG antibody and anti-tubulin antibody as a loading control. Data are normalized to protein levels at time 0. All results are mean ± s.e.m, n = 4.

The data in Figure 2 indicate that cells and tissues are able to express the splice variant, but no truncated SULT4A1 protein was detected. We therefore asked whether protein produced from each of the mRNAs was stable. Constructs encoding both FLAG-tagged wild-type and truncated SULT4A1 produced protein when ectopically expressed in IMR-32 cells (Fig. 4). The stability of the expressed proteins was determined by treating the transfected cells with cycloheximide. Figure 4 shows that the wild-type SULT4A1 was stable over 8 hr with only a 20% decrease in protein levels. By contrast, the truncated protein from the splice variant transcript was rapidly degraded with a half-life of 2–3 hr. These results indicate that the splice variant mRNA can be translated, but the resulting protein is unstable *in vivo*.

### Dimerization of SULT4A1

**Figure 5:**
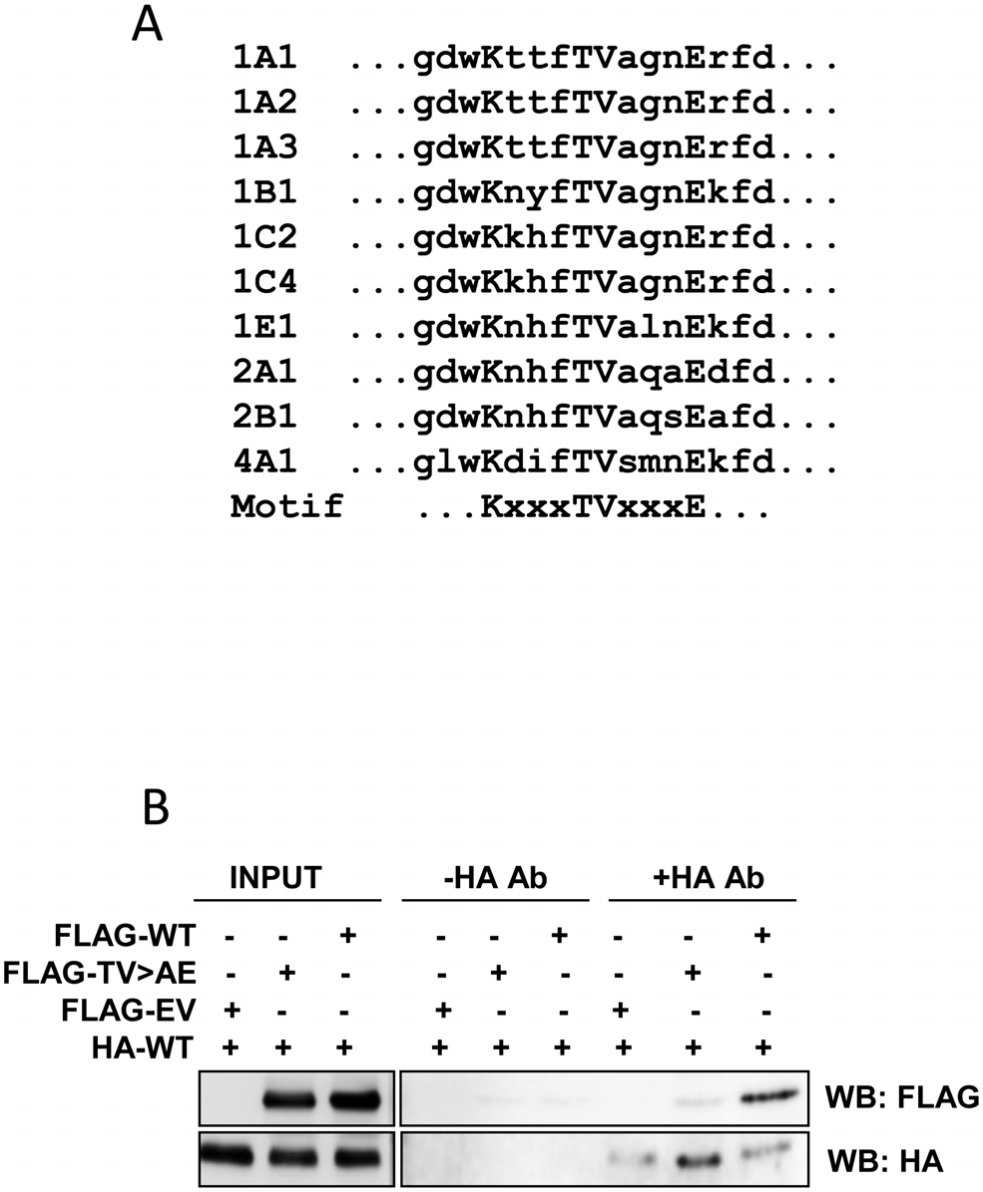
Homodimerization of SULT4A1. (A) Conservation of the dimerization motif in various human SULTs. The consensus sequence is shown below. (B) Immunoprecipitaton assay for dimerization of SULT4A1 protein. IMR-32 cells were co-transfected with HA-tagged SULT4A1 and either FLAG-tagged wild-type SULT4A1 (WT), mutant SULT4A1 (TV) or empty vector (EV). Isolated cytosolic proteins were immunoprecipitated with anti-HA antibody and detected with anti-FLAG antibody. Western blots of protein lysates prior to immunoprecipitation (Input) demonstrate expression. Western blots using anti-HA antibody (lower panel) demonstrates immunoprecipitation.

Almost all SULTs contain a conserved motif (KxxxTVxxxE) that has been shown to be necessary for the formation of sulfotransferase dimers [15]. Crystallographic studies show a head-to-tail arrangement of this motif and suggest binding involves hydrogen bonds between the conserved amino acids [16]. The wild-type SULT4A1 contains the dimerization motif, although it shares the least homology with other human sulfotransferases (Fig. 5A). Interestingly, SULT4A1 lacks the conserved alanine adjacent to the central threonine-valine (TV). In human SULT2A1, a nonsynonymous SNP in the codon for this alanine has been reported in 13% of African-Americans. The mutation, which changes the alanine to a threonine, disrupts dimerization [17]. To test whether SULT4A1 is homomeric or dimeric, FLAG-tagged and HA-tagged SULT4A1 protein was expressed in IMR-32 cells. Immunoprecipitation showed dimerization of the different tagged proteins (Fig. 5B). Moreover, when the central TV in the dimerization motif was mutated to AE, homodimerization of SULT4A1 was inhibited. Taken together, these results indicate that SULT4A1 homodimerizes similarly to most other human sulfotransferases.

### Dimerization Inhibits Polyubiquitination

**Figure 6:**
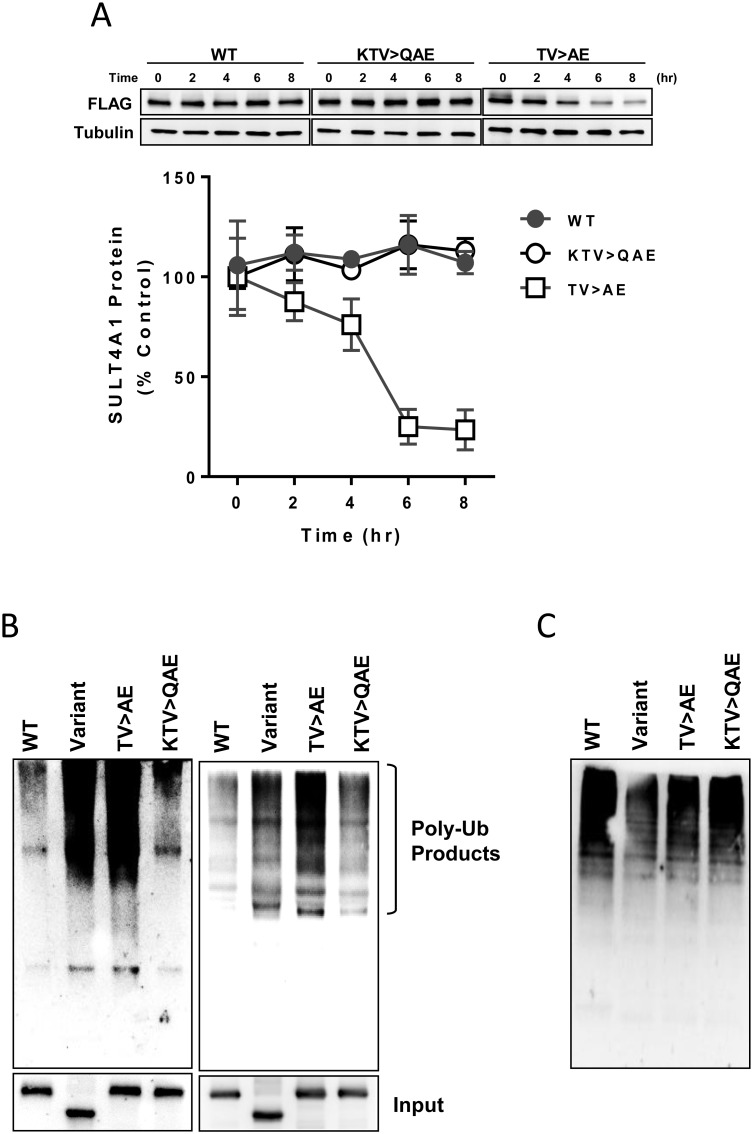
Role of dimerization in SULT4A1 stability. (A) IMR-32 cells transfected with wild-type (WT), TV>AE mutant or KTV>QAE mutant SULT4A1 were treated with 10 µg/ml cycloheximide and protein was collected at the different times. Western blots of protein were quantified by densitometry and normalized to tubulin as a loading control. Data are expressed relative to protein levels at time 0 (% Control). All results are mean ± s.e.m, n = 3. (B) Polyubiquitination of SULT4A1 wild-type and mutant proteins. IMR-32 cells were transfected with the different SULT4A1 FLAG-tagged constructs along with a HA-tagged ubiquitin construct. Following treatment with 20 µM MG132 for 8 hr, Western blots of HA-tagged proteins were performed. Expression of each construct was confirmed with anti-FLAG antibody (Input). Left panel represents immunopreciptation before protein denaturation while the right panel represents immunoprecipitation after protein denaturation. (C) Western blot for HA-ubiquitin on supernatants prior to immunoprecipitation.

The physiological significance of sulfotransferase dimerization has been examined for a number of different enzymes. For SULT2A1, dimerization affects enzyme kinetics, substrate binding to the allosteric site and substrate inhibition [18]. By contrast, the homomeric form of SULT1A1 is structurally unstable and is more readily denatured by temperature and urea compared to the wild-type protein [19]. Here, we examined the stability of the wild-type dimeric form of SULT4A1 and the TV>AE mutant, which does not form dimers. Similar to that shown in Figure 4, the wild-type protein was stable over 8 hr in cells treated with cycloheximide (Fig. 6A). By contrast, the TV>AE mutant disappeared with a half-life of approximately 5 hr. By 8 hr, less than 25% of the protein remained. The instability of the mutant protein suggested that dimerization may prevent intracellular degradation similar to other protein systems such as p62 scaffold protein [20], nitric oxide synthase [21] and C/EBP transcription factors [22]. For some of these proteins, access to polyubiquitination sites by ubiquitin ligases is sterically hindered.

The lysine located in the conserved dimerization site of the sulfotransferases is a potential polyubiquitination site so we mutated it to a glutamine and determined protein stability *in vivo*. Figure 6A shows that this mutation prevented degradation of the unstable monomeric SULT4A1 (TV>AE). We also performed a polyubiquitination assay on the wild-type and the mutant SULT4A1 proteins. While the wild-type protein showed very little polyubiquitination (Fig. 6B, left panel, lane 1), the unstable TV>AE mutant was extensively ubiquitinated (Fig. 6B, left panel, lane 3). The polyubiquitinated products was not due to potential binding partners as a similar polyubiquitination pattern was seen when the SULTs were denatured before immunoprecipitation (Fig. 6B, right panel). In addition, the variance in polyubiquitination was not due to differences in Ha-tagged ubiquitin expression (Fig. 6C).

Mutation of the lysine located in the dimerization motif significantly inhibited polyubiquitination (Fig. 6B, left panel, lane 4). Finally, the unstable truncated SULT4A1 protein expressed from the splice variant mRNA was also extensively polyubiquitinated, consistent with its lack of dimerization motif and lack of stability *in vivo* (Fig. 6B, left panel, lane 2).

## Discussion

Using northern blot analysis, early work on the cloning and expression of SULT4A1 reported that mRNA for the gene was only detectable in human and rat brain [4]. More recent studies in mice using PCR confirmed the high level of expression in brain tissue, but also detected lower expression in the liver and GI tract [5]. Consistent with this, we have shown in the present study that SULT4A1 may be expressed in a number of tissues as well as the brain in humans. Surprisingly, we observed the presence of the splice variant in many tissues. Some, such as the small intestine, colon and prostate expressed both transcripts, although it is not possible from this study to discern if these are localized to the same cell type. We also observed expression of the variant transcript in most cell-lines with some (PC-12 and SH-SY5Y cells) expressing both. Interestingly, the variant transcript was readily detectable even though it has been suggested that it may be unstable and degraded by nonsense mediated mRNA decay. In both SK-N-MC and SH-SY5Y cells, differentiation into neurons resulted in a switch from the variant transcript to the wild-type, which was accompanied by increased expression of the SULT4A1 protein. This may represent a post-transcriptional mechanism for regulating SULT4A1 expression. Transcriptome diversity has been shown to arise from both alternative transcription of genes as well as alternative splicing of mRNA [23]. Recently, switching between transcripts by alternative splicing during embryonic development and cell differentiation has been widely reported [24], [25], [26]. In mice, SULT4A1 transcripts are present in embryonic, fetal and adult brain tissue. It would be interesting to see if the variant transcript is present at any time throughout development and whether switching between transcripts occurs.

Although SULT4A1 mRNA was present in numerous cells and tissues, the protein was only detected in those cells where the wild-type transcript was expressed. This suggested that the variant transcript was not translated or that the truncated protein was rapidly degraded. Ectopic expression confirmed that the variant transcript could be translated, but the resultant protein was unstable. We also found that the truncated protein was highly polyubiquitinated, which is consistent with its rapid degradation. Premature stop codons that result is C-terminus truncation commonly lead to misfolded proteins that are targeted for degradation [27].

Similar to other cytosolic sulfotransferases, SULT4A1 homodimerizes through the conserved motif located towards the C-terminus of the protein [3]. This motif is found in most SULTs, although it is not present in murine SULT1E1. The exact role for dimerization remains unknown, but several recent studies have shown significant functional or structural differences between the monomeric and dimeric forms of various SULTs. For example, Thomae et al [17] reported that a naturally occurring mutation in the dimerization motif of SULT2A1 (A261T) resulted in monomeric protein with similar capacity to metabolize dehydroepiandrosterone as the wild-type protein. However, Cook et al [18] showed using a fusion protein of maltose binding protein and SULT2A1 that the monomeric form of the enzyme did not exhibit the typical substrate inhibition seen with the dimeric form. Similarly, human SULT1E1, which is a homodimer, exhibits strong substrate inhibition [28] while murine SULT1E1, which is a monomer, does not [29]. For human SULT1E1, only one subunit can form a product during a single catalytic cycle suggesting the subunits of the dimer do not behave independently [30]. Dimerization has also been shown to stabilize SULT1A1 during thermal and chemical denaturation [19]. Here, mutation of the SULT4A1 dimerization motif inhibited dimerization and enhanced degradation of the resulting protein. The instability was due to polyubiquitination, similar to that seen with the truncated protein. Interestingly, when we further mutated the conserved lysine in the dimerization motif, polyubiquitination was inhibited and the protein showed similar stability to wild-type SULT4A1. These results suggest that one function of SULT dimerization is to mask a polyubiquitination site, at least for SULT4A1, in a similar manner as that described for neuronal nitric oxide homodimers [21] and PEBPsβ-RUNX1 heterodimers [31]. The dimerization motif for the SULTs is a hydrophobic interface with two salt bridges at either end [16], which would be exposed when dimer formation is inhibited. Hydrophobic patches are recognized by chaperones involved in protein quality control and recruit ubiquitin ligases that mark the protein for degradation [32]. It would be interesting to extend these findings to other SULTs by determining the stability of the homomeric forms *in vivo*.

In conclusion, SULT4A1 is an orphan sulfotransferase that is widely expressed, although primarily as a variant transcript that does not result in measurable protein expression. The relationship between the variant and wild-type transcript deserves further study, particularly as there is the capacity to switch from one transcript to the other. This may be an important post-transcriptional regulatory mechanism for the protein. Finally, dimerization of SULT4A1 was demonstrated, and is responsible for the stability of the protein within the cell. While the precise biological function for SULT4A1 remains to be determined, its intracellular regulation [7], [8] and stability appears to be finely controlled.
